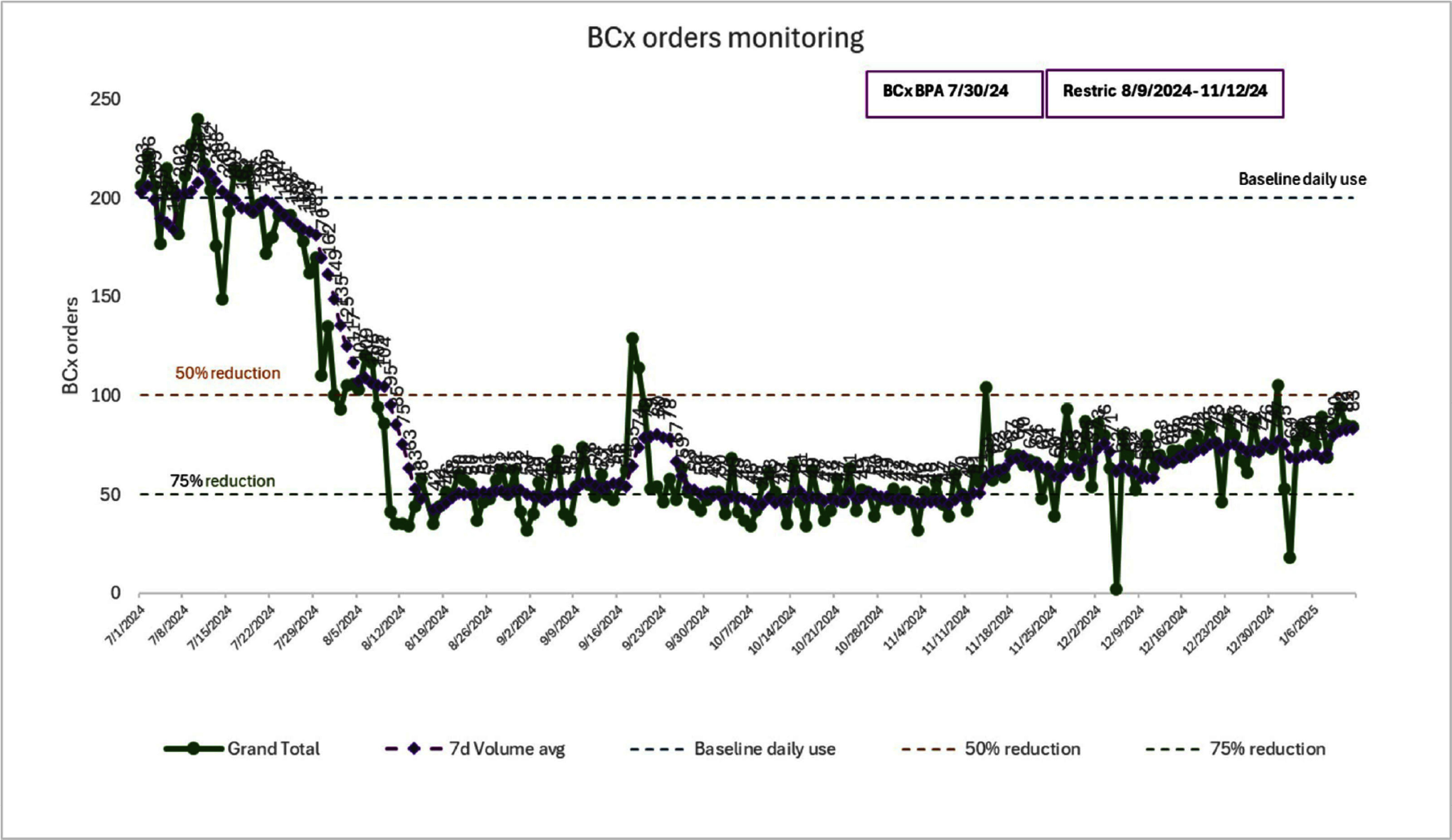# Sustained Impact of EMR Best Practice Guidance on Blood Culture Bottle Utilization

**DOI:** 10.1017/ash.2025.323

**Published:** 2025-09-24

**Authors:** Inessa Gendlina, Adam Zimilover, Gregory Weston, Ruchika Jain, Priya Nori, Marilou Corpuz, Erika Orner, Sammy Cheng, Julia Piwoz, Wendy Szymczak

**Affiliations:** 1 Einstein Montefiore; 2 Montefiore Medical Center - Albert Einstein College of Medicine; 3 Montefiore Medical Center and Health System Bronx NY; 4 Montefiore Health System; 5 Montefiore Medical Center; 6 Albert Einstein College of Medicine; 7 Montefiore Medical Center

## Abstract

**Background:** Blood cultures are essential for the accurate diagnoses of sepsis and bacteremia and have been recommended and used liberally as part of the diagnostic workup. Previous studies have shown that judicious use of blood cultures is safe in both adults and children (PMID 31942949). In the summer of 2024, BD BACTEC experienced a national shortage of blood culture media bottles, prompting institutions nationwide to implement measures to conserve supplies. Our institution rapidly implemented a blood culture diagnostic stewardship program, adopting a tiered approach including refining guidelines for blood culture orders via institution-wide education, and leveraging EMR both via best practice advisories (BPAs) on appropriate culturing and ordering restrictions. In this study, we evaluate the impact of these interventions and the post-restriction effects of EMR-based education. **Methods:** Prior to the shortage, no clinical decision support existed in the EMR to guide the blood culture ordering process. Initial measures implemented in July 2024 included a BPA highlighting appropriate indications for ordering initial and follow-up blood cultures (PMID 39136555; ASM Blood Culture Bottle Inventory Management and Clinical Conservation During Supply Shortages). In August 2024, restrictions were introduced, limiting orders to one set per 72 hours, with case-by-case overrides managed by an Infectious Diseases-led diagnostic stewardship team. After supplies improved in November 2024, restrictions were lifted, but the BPA-based clinical guidance was retained. Blood culture volumes were monitored across three phases: pre-shortage, during restrictions, and post-restriction. **Results:** Blood culture volume decreased by approximately 50% immediately following the introduction of BPAs and further decreased to 75% of pre-shortage levels during the restriction period. Post-restriction, at 2 months follow-up, culture volume has stabilized and sustained at over 50% lower than pre-shortage levels. **Conclusion:** The implementation of in-EMR best practice guidance, and temporary restrictions during a blood culture media shortage, led to significant reductions in blood culture order volume, even after lifting restrictions. These findings support the role of diagnostic stewardship interventions in promoting lasting changes in provider behavior. Utilizing EMR to support best practices and aligning blood culture practices with evidence-based indications, can reduce unnecessary testing and improve resource utilization. Further research is needed to evaluate the impact of these changes on patient outcomes.

**Figure:**